# Prediction of Autoimmune Diseases by Targeted Metabolomic Assay of Urinary Organic Acids

**DOI:** 10.3390/metabo10120502

**Published:** 2020-12-08

**Authors:** Dimitris Tsoukalas, Vassileios Fragoulakis, Evangelos Papakonstantinou, Maria Antonaki, Athanassios Vozikis, Aristidis Tsatsakis, Ana Maria Buga, Mihaela Mitroi, Daniela Calina

**Affiliations:** 1 Department of Clinical Pharmacy, University of Medicine and Pharmacy of Craiova, 200349 Craiova, Romania; 2 Metabolomic Medicine, Health Clinic for Autoimmune and Chronic Diseases, 10674 Athens, Greece; vagpap25@gmail.com; 3 European Institute of Nutritional Medicine (E.I.Nu.M.), 00198 Rome, Italy; 4 The Golden Helix Foundation, London WC2N 5AP, UK; vfragoulakis75@gmail.com; 5 Laboratory of Health Economics & Management, Economics Department, University of Piraeus, 18534 Piraeus, Greece; enmarry1993@yahoo.gr (M.A.); avozik@unipi.gr (A.V.); 6 Laboratory of Toxicology and Forensic Sciences, Medical School, University of Crete, 71003 Heraklion, Greece; tsatsaka@uoc.gr; 7 Department of Analytical and Forensic Medical Toxicology, Sechenov University, 119991 Moscow, Russia; 8 Department of Biochemistry, University of Medicine and Pharmacy of Craiova, 200349 Craiova, Romania; ana.buga@umfcv.ro; 9 ENT Department, University of Medicine and Pharmacy of Craiova, 200349 Craiova, Romania; mihaela.mitroi48@gmail.com

**Keywords:** autoimmune diseases, metabolomics, organic acids, tricarboxylate cycle, glutathione cycle, disease prediction, artificial intelligence

## Abstract

Autoimmune diseases (ADs) are chronic disorders characterized by the loss of self-tolerance, and although being heterogeneous, they share common pathogenic mechanisms. Self-antigens and inflammation markers are established diagnostic tools; however, the metabolic imbalances that underlie ADs are poorly described. The study aimed to employ metabolomics for the detection of disease-related changes in autoimmune diseases that could have predictive value. Quantitative analysis of 28 urine organic acids was performed using Gas Chromatography-Mass Spectrometry in a group of 392 participants. Autoimmune thyroiditis, inflammatory bowel disease, psoriasis and rheumatoid arthritis were the most prevalent autoimmune diseases of the study. Statistically significant differences were observed in the tricarboxylate cycle metabolites, succinate, methylcitrate and malate, the pyroglutamate and 2-hydroxybutyrate from the glutathione cycle and the metabolites methylmalonate, 4-hydroxyphenylpyruvate, 2-hydroxyglutarate and 2-hydroxyisobutyrate between the AD group and the control. Artificial neural networks and Binary logistic regression resulted in the highest predictive accuracy scores (66.7% and 74.9%, respectively), while Methylmalonate, 2-Hydroxyglutarate and 2-hydroxybutyrate were proposed as potential biomarkers for autoimmune diseases. Urine organic acid levels related to the mechanisms of energy production and detoxification were associated with the presence of autoimmune diseases and could be an adjunct tool for early diagnosis and prediction.

## 1. Introduction

Autoimmune diseases (ADs) are a diverse group of chronic disorders, including rheumatoid arthritis (RA), Hashimoto’s thyroiditis (HT), psoriasis (PSO), vitiligo (VIT) and inflammatory bowel diseases (IBD) caused by the loss of tolerance to self by the immune system and currently affect 5–10% of the population [1].

ADs can be organ-specific or systemic, leading to different health complications and disabilities. Prevalence rates for ADs can vary due to the high diversity of the group of ADs and the constantly new conditions that are added in the category of ADs and related conditions [2]. As stated in the report on Autoimmune Diseases Workshop of the European Parliament, data on ADs epidemiology is insufficient and limited to only some of the Ads, providing only a part of the picture [3].

ADs are a major public health problem because they are often accompanied by musculoskeletal problems that deteriorate the quality of life, accounting for a significant number of Disability Adjusted Life Years (DALYs) lost due to the condition, having a great economic and mental impact [4,5]. ADs rarely have one manifestation, but they are rather combined with other types of Ads. For example, patients with autoimmune thyroiditis (Grave’s or Hashimoto’s diseases) have at least one more AD, with RA being the most prevalent [6], or cardiometabolic complications further aggravating the disease [7].

ADs share common features and molecular pathways that are linked to the loss of self-tolerance from the immune system. Dysregulated immune responses characterized by increased auto-reactive T cells and reduced regulatory T cells lead to the non-resolving low-grade chronic inflammation, which is a hallmark of ADs. Depending on the type of ADs, different autoantigens have been recognized, facilitating the diagnosis of the disease [8]. Diagnosis of an AD can be time-consuming and expensive, given that ADs can manifest with many different symptoms requiring the consultation of several different specialists before reaching a diagnosis, especially when it can be triggered by a specific treatment, for example antiviral treatments [9].

Another important issue of ADs is that they are characterized by relapses and remissions, which manifest as increases and decreases in the immune response markers, including TNFα [10,11]. As a consequence, studies aiming to identify biomarkers that can monitor disease severity or treatment efficacy should depend on mechanisms that provide a systemic overview of the cells and organism as a biological system rather than a located immune response.

Metabolomics has attracted increasing attention in the field of biomarker discovery because it captures the interaction of genes and environmental triggers that is expressed at a given time, thus can have clinical application. Additionally, sample collection requires the minimum level of intervention as it can be performed in urine or blood in addition to a more location-specific site such as Cerebrospinal fluid (CSF) [12]. Last but not least, metabolomics is a low-cost method allowing repeated measurements in a short time, providing close monitoring of the metabolic state of the patient in response to disease and treatment adjustments [13].

Organic acids (OAs) are intermediate metabolites of critical cellular metabolic pathways, including but not limited to the energy production pathway in the mitochondria via citric acid or tricarboxylate (TCA) cycle, metabolism of carbohydrates and proteins, ketone bodies’ metabolism and other related pathways [14]. Additionally, selected OAs have been linked to the microbiome status, antioxidant capacity, metabolism of neurotransmitters and vitamin bioavailability. In addition, OAs can provide valuable information on the nutritional and vitamin adequacy, metabolism of drugs, and microbiome unbalances [15]. Previous studies analyzing organic acids in autoimmune diseases have significantly contributed to the biomarker discovery, though the limited number of studies and the lack of repeated findings have hampered their validation [16].

The aim of the present study was to identify metabolic changes in the OAs of individuals with ADs and develop predictive algorithms for the presence of ADs through the integration of metabolomics and artificial intelligence (AI).

We have performed targeted metabolomics using Gas Chromatography-Mass Spectrometry in a case-control exploratory study of prevalent ADs (RA, THY, PSO, VIT, IBD, MS,) and other less prevalent (OTHER). Correlation and pathways analysis demonstrated metabolite-metabolite correlations and inter-pathway changes in ADs, and a predictive algorithm was developed to estimate the predictive probability of ADs presence based on the OAs profile.

## 2. Results

### 2.1. Distinct Levels of Organic Acids in ADs

Targeted metabolomic analysis was performed on 28 urinary organic acids, and the absolute concentrations for both arms are shown in Table 1.

**Figure 1:**
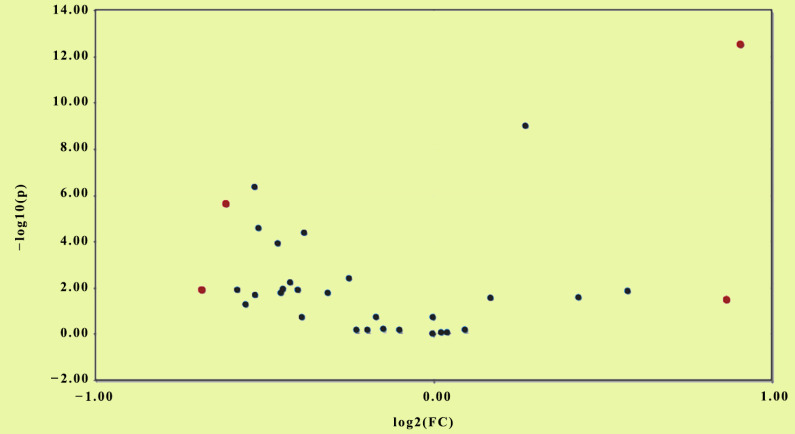
Volcano plot of variables between AD and control groups (False Discovery Rate (FDR) < 0.05, fold change (FC) > 1.5). Red dots indicate significantly different variables in relation to control.

**Figure 2:**
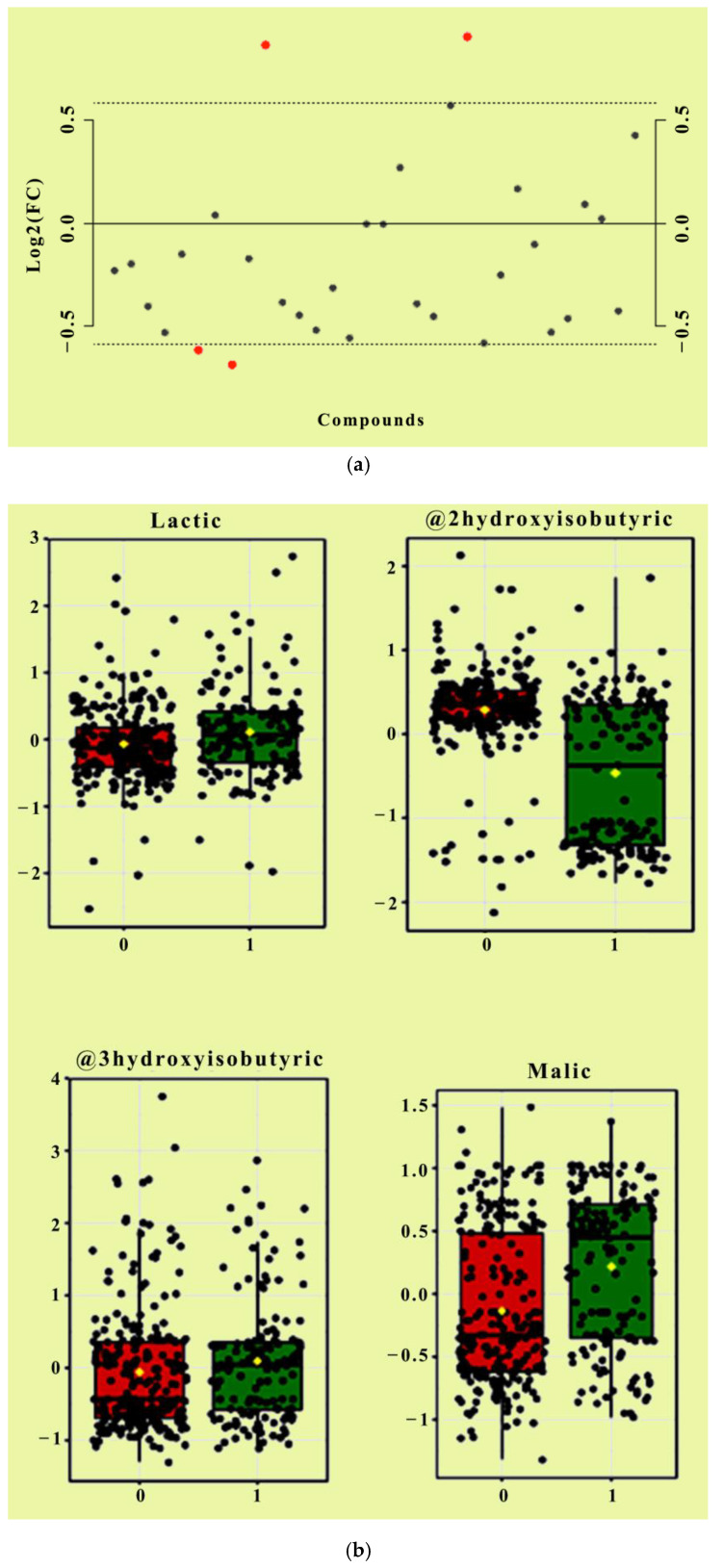
Fold Change Analysis of the organic acid metabolic compounds between the two groups (FDR < 0.05, fold change (FC) > 1.5). Red dots indicate significantly different variables in relation to control (**a**). Bar plots show the untransformed values (mean ± SD) where “1” indicates the control group (red) and “0” indicates the AD group (green) (**b**).

Values of mean ± SD, Median and *p* values after Bonferroni correction are demonstrated for the 28 organic acids. Differences between two groups were assessed using the non-Parametric Mann- Whitney test since Q-Q plots indicated that distributions between groups do not follow the normal distribution. Bonferroni correction was applied, and statistically significant differences are shown in bold. A total of nine metabolites were markedly different between the two groups. Specifically, succinic acid, malic acid, pyroglutamic acid, methylmalonic acid, 2-hydroxybutyric acid, methylcitric acid and 4-hydroxy phenylacetic acid were decreased in cases compared with the other arm. Conversely, 2- hydroxyglutaric acid and 2-hydroxyisobutyric acid were statistically significantly increased in the group with patients with AD compared to the control. Boxplots of the levels of the main variables used in the analysis are presented in Figure S3 for both groups. In addition, a False Discovery Rate analysis (FDR) was conducted to adjust the *p*-values of the multiple correlations among the variables. The analysis revealed that 4 out of 28 compounds were significantly regulated (FDR < 0.05, fold change (FC) > 1.5) (Figure 1 and Figure 2), including lactic acid, 2-hydroxybutyric acid, 3-hydroxybutyric acid and 2-hydroxyisobutyric acid, and were found significant after FDR adjustment. Since a certain proportion of samples was reported to be below the limit of detection (LOD), known as left censored data, descriptive statistics analysis was also performed by replacing left censored values, producing similar results (Table S2). Among existing statistical approaches to correct this type of bias under certain assumptions, in the present study, it was realistically assumed that the data were righted skewed, and thus, the distribution of interest was log-normal. Because the magnitude of skewness is not known a-priori, although important for the final judgment, a sensible approach with desired statistical properties was to be replaced each censored value with LOD/√2 (LOD = 1 mmol/mol Creatinine) [17].

**Figure 3:**
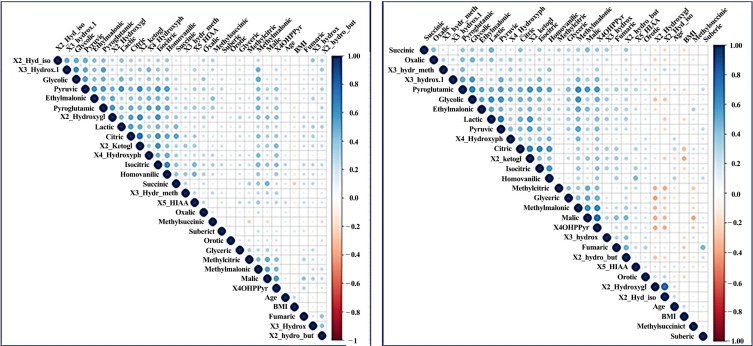
A scatter plot correlation matrix of the main variables used in the model. (Left) AD group (Right) Control group. Positive correlations are shown in blue and negative correlations are shown in red. Abbr: X2_Hyd_iso: 2-Hydroxyisobutyric acid, X3_hydrox.1: 3 hydroxyisovaleric acid, X2_Hydroxygl: 2-Hydroxyglutaric acid, X2_ketogl: 2-Ketoglutaric acid, X4_Hydroxyph: 4-Hydroxyphenylacetic acid, X3_hydr_meth: 3-Hydroxy-methylglutaric acid, X5_HIAA: 5-Hydroxyindoloacetic acid, X4OHPPyr: 4-Hydroxyphenypyruvic acid, X3_hydrox: 3-Hydroxybutyric acid, X2_hydro_but: 2-Hydroxybutyric acid.

Spearman rho analysis was performed to identify the statistically significant correlations. A total of 496 correlations were identified, and 289 correlations were significant (*p* < 0.05). Specifically, the correlation analysis was performed in 28 organic acids and four demographic and lifestyle factors, including age exercise, alcohol consumption and BMI, namely 32 variables in total. The mathematical formula which calculates the number of pairwise correlations was: Total number= *n* × (*n* − 1)/2, where *n* = 32, hence 496 comparisons. Figure 3 depicts the positive (blue) and negative (red) correlations for the two groups. In both groups, there were 64 negative correlations, but with no statistical significance. The strongest metabolite-metabolite correlation was limited to 0.597 between Citric and 2-ketoglutaric acid. Age was correlated to citric, isocitric, Homovanillic, 5-HIAA, 4 Hydroxyphenylacetic, 2-hydroxy isobutyric and ethylmalonic organic acids.

The baseline characteristics of the participants are demonstrated in Table S3. Age, gender and BMI did not differ between the two groups (*p* > 0.05). Females were 69.5% of the cases, and 62.9% of the healthy participants and the respective mean ± SD levels of BMI were 25.3 ± 4,7 and 25.0 ± 4.1. Cross comparison between participants with AD and control for their levels of physical activity and alcohol consumption demonstrated that they were both higher in the AD group (*p* < 0.001). Among patients with ADs, the most common AD was autoimmune thyroiditis (THY), accounting for 53% of the case followed by IBD (18.5%), PSO (15.22%), MS (9.6%), RA (6.6%), VIT (4.5%) and AD (0.2). The group OTHER accounted for 22.6% of the cases, but only 6.6% of them had OTHER AD alone with no other comorbidity of the selected ADs and included a variety of less common ADs listed in Table S1. The frequency of comorbidity among cases was 27.1%.

### 2.2. Pathways Analysis

**Figure 4:**
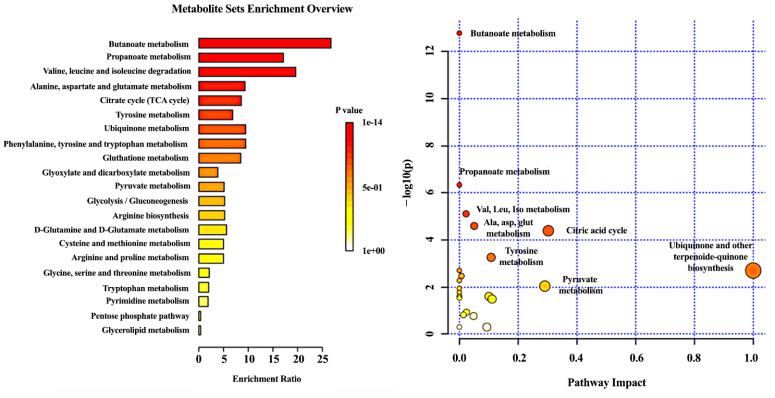
Enrichment analysis (**Left**) and pathway topology analysis (**Right**) of altered metabolic pathways in AD both obtained from MetaboAnalyst. In the pathway analysis diagram *X* axis indicates the impact of selected metabolites in the presented pathway while the *Y* axis shows level of enrichment of the pathway.

A total of 28 organic acids analyzed were involved in 21 metabolic pathways. Figure 4 shows the bar chart of pathway enrichment analysis depicting the metabolic pathways that were affected in ADs based on quantitative enrichment analysis (QEA). Analysis showed that 16 out of the 21 pathways reached statistical significance in terms of *p*-value and FDR values. Butanoate metabolism was the most affected metabolic pathway (*p* < 0.0001, FDR < 0.0001), in which four metabolites were included in our selected panel of metabolites, namely, succinic acid, 2-ketoglutaric acid, 2-hydroxyglutaric acid and 3-hydroxybutyric acid. Other significantly affected pathways were the propanoate metabolism, the valine, leucine, isoleucine metabolism, the alanine, aspartate and glutamate metabolism and the phenylalanine, tyrosine and tryptophan metabolism. In addition, QEA showed that the citric acid cycle was significantly disturbed, where 6 of 20 metabolites were included in our panel, followed by the tyrosine metabolism with 5 out of 42 hits and the glutathione metabolism. Additionally, the impact of metabolites on the pathway was assessed, and the ubiquinone and other terpenoid-quinone biosynthesis had the highest impact, a pathway where 4-hydroxyphenylpyruvate participates, followed by the TCA cycle and the pyruvate metabolism.

### 2.3. Development of Predictive Models

A straightforward binary logistic regression model was used to assess the association of the presence of ADs with selected OAs and other parameters, and results are shown in Table 2.

**Figure 5:**
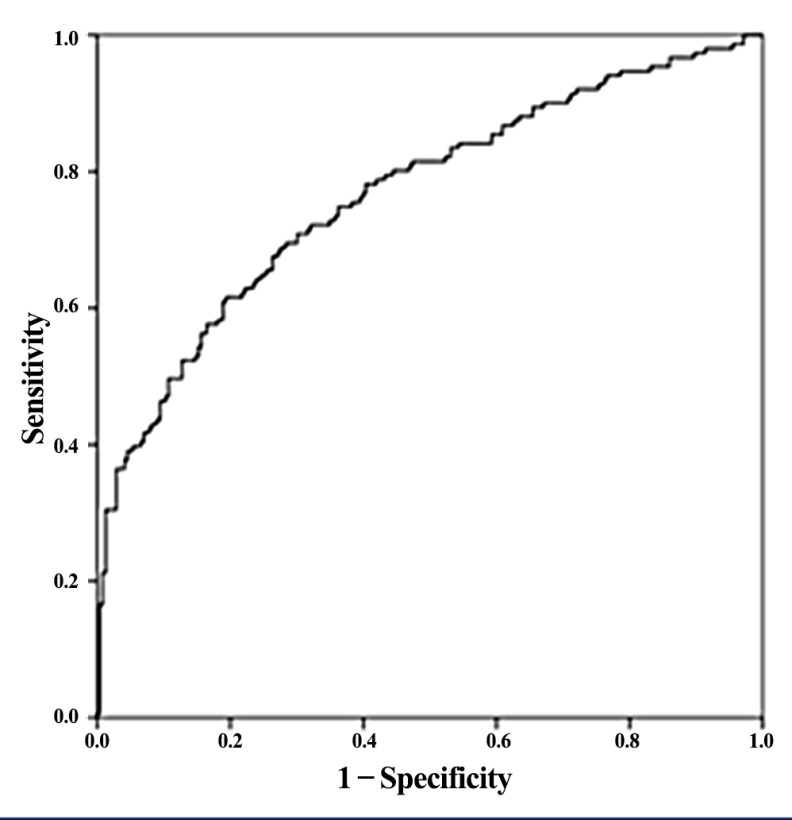
ROC Curve for the Logistic Model.

The variables included were statistically significantly different in the case group compared to the control. Following results, 2-hydroxyisobutyric were negatively correlated with the absence of an AD, while 2-hydroxybutyric was positively correlated. Lack of exercise and alcohol consumption was positively correlated with the presence of an AD. The Hosmer and Lemeshow (H-L) goodness of fit test was X^2^ (8) = 9.585, *p* = 0.295, and the (pseudo) R^2^, Nagelkerke test, was equal to 0.283. The predictive accuracy was 90.41% and 50.3% for patients with ADs and the control group, respectively, whereas the model was able to predict with 74.9% accuracy for both groups. As depicted in Figure 5, the area under the curve (ROC) was estimated at 0.767.

A Principal Component Analysis (PCA) was performed with a relatively good sampling adequacy of 0.819 estimated by Kaiser-Meyer-Olkin, while a statistically significant Bartlett’s test of sphericity was found (X^2^ (435) = 3.464, *p* < 0.001). The first ten components were able to explain 63% of the variance in total. Less than 3.0% of the total variance was explained by each of the other components (Table S4). Hence, the ten-component model was applied, with eigenvalues greater or equal to 1. The ten-factor PCA model was assessed using a binary logistic regression model, and the results are shown in Table 3. A negative association was found between the log of the odds of the presence of ADs and components four, seven, eight and ten, but only factor four was statistically significant. (*p* < 0.001). The (H-L) and Nagelkerke (pseudo) R^2^ tests were estimated at X^2^ (8) = 8655 with a *p*-value equal to 0.372 and R^2^ equal to 0.281, respectively. The binary logistic regression model using the principal components was able to identify individuals belonging to the case group with 84.3% accuracy and the control group with 49.3%. The overall predictive accuracy of the model was estimated at 66.8%.

**Figure 6:**
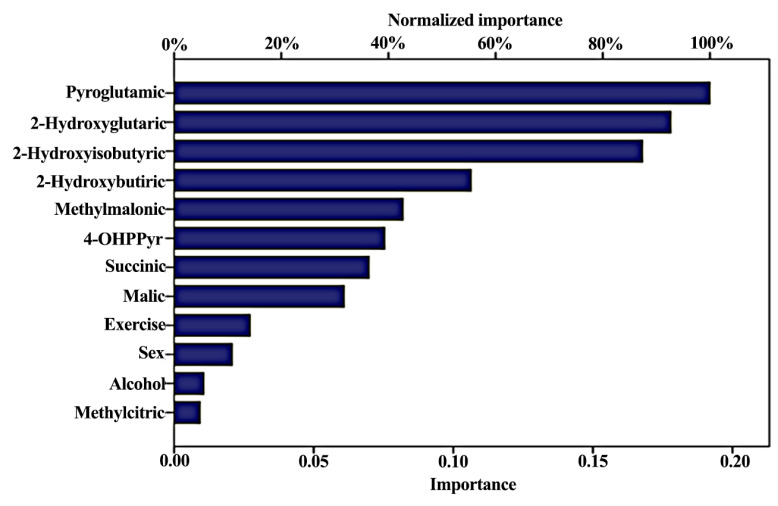
Contribution of Organic Acids and demographic characteristics to the predicted accuracy of the Artificial Neuronal Networks (ANN).

Artificial Neuronal Networks (ANN) analysis was employed as a predictive model for the presence of ADs based on OAs and other patient variables. An exploratory methodology was applied to our dataset with different architectures. Due to our relatively small sample size and to avoid overfitting, the model was reduced one hidden layer, and 12 variables were used. The training dataset had 271 (68.8%) observations, while the test set had 72 observations (18.3%), and there were 51 observations (12.9%) as a holdout. The model parameters of the ANN are shown in Table S5. ANN predicted those with an AD with 92.6% predictive accuracy. The total predictive value of the model reached 66.7 for both groups, as presented in Table 4. ROC analysis resulted in an 0.88 area under the curve for both arms. Of the analyzed variables, Methylmalonic, 2-Hydroxyglutaric and 2-hydroxy butyric were important markers for the ANN model (Figure 6).

## 3. Discussion

Metabolomics is an emerging tool for the prediction and early diagnosis of autoimmune diseases since it can capture metabolic changes that are associated with the presence of, or predisposition to a disease.

In the present study, we have quantitatively assessed the organic acids profile of patients with ADs, based on which we developed predictive models that reached 92.6% accuracy for patients with AD(s). Previously, we have shown that the integration of artificial intelligence with metabolomics analysis of fatty acids can identify metabolic biomarkers associated with the presence of ADs [18].

**Figure 7:**
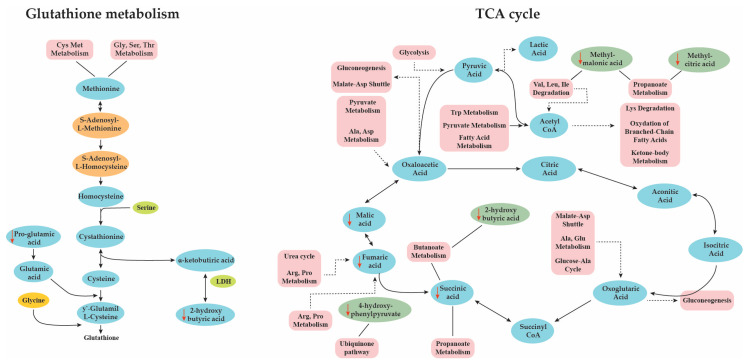
Summary of significantly altered organic acids in ADs and involved pathways.

Comparative analysis of urine OAs in patients with ADs and control demonstrated statistically significant differences in succinic acid, malic acid, pyroglutamic acid, methylmalonic acid, 2-hydroxyglutaric acid, 2-hydroxyisobutyric acid, 2-hydroxybutyric acid, methylcitric acid and 4-hydroxyphenylpuryvic acid, which remained significant after the Bonferroni correction (Table 1). False discovery rate (FDR) analysis was also performed to identify the affected metabolites after adjusting for multiple corrections and lactic acid, 2-hydroxyisobutyric acid, malic acid, and 3-hydroxybutyric acid reached statistical significance (FDR < 0.05, fold change (FC) > 1.5). Succinic acid and malic acid are key components of the TCA cycle, and in our results, they were markedly decreased in the ADs group compared to the control. Notably, all the metabolites of the TCA cycle were downregulated in the ADs group (citric acid, isocitric acid, oxoglutaric acid, fumaric acid and oxalic acid) even though these differences did not reach statistically significant levels. The TCA cycle is the central metabolic pathway network of the cells for the production of energy. The TCA cycle is fueled by the catabolism of macronutrients (amino acids, carbohydrates, lipids) and ketone metabolism. At the same time, TCA intermediate metabolites serve as substrates for other metabolic networks, the majority of which are summarized in Figure 7 [14].

In our study, levels of TCA intermediate metabolites were lower in the AD group compared to the control, suggesting reduced energy production and disrupted fueling of the TCA-linked pathways. A possible explanation of these findings would be the consumption of nutrient-empty foods due to poor dietary habits or reduced intake due to pain and discomfort, leading to insufficient intake of micronutrients, which act as co-factors in metabolism. On the other hand, even if nutrient-dense foods are consumed, malabsorption is very common among patients with ADs and particularly IBD and other ADs with gastrointestinal complications. Vitamins and other micronutrients are not effectively absorbed in the gastrointestinal tract resulting in reduced transport and use in the metabolism.

Levels of pyroglutamic acid or 5-oxoproline, a metabolite of the glutathione cycle that is converted to glutamate by 5-oxoprolinase, were statistically significantly lower in the group of ADs compared to the control. Reduced levels of pyroglutamic acid could indicate low glutathione recycling caused by the insufficiency of dietary amino acids that are required for glutathione synthesis or high glutathione depletion due to the upregulation of detoxification mechanisms [19]. Pyroglutamic acid is also important for free amino acid transportation, and lower pyroglutamic acid levels have been associated with type 2 diabetes and increased glucose levels [20,21].

2-hydroxybutyric or a-hydroxybutyric acid is naturally produced from the conversion of a-ketobutyrate or 2-oxobutanoate as a byproduct in the anabolism of glutathione when cystathione is converted to cysteine. The production of a-ketobutyrate derives from the degradation of methione and threonine. (Figure 7). 2-hydroxybutyric acid mainly originates in hepatic cells and reflects the glutathione synthesis flow in conditions of metabolic or oxidative stress, while it has been suggested as an early marker for the evaluation of insulin resistance and impaired glucose levels regulation. In our study, 2-hydroxybutyric acid was markedly decreased in patients with ADs compared to the control, in line with the changed levels of pyroglutamic acid. These findings show a significant disruption of the glutathione cycle and possibly reduced glutathione synthesis and reduced detoxification capacity.

Methylmalonic acid (MMA) is a downstream metabolite of MMA-CoA, participating in the metabolic pathways of vitamin B12 or cobalamin, and is a known marker for Vitamin B12 bioavailability [22]. In our study, methylmalonic acid was found to be markedly decreased in patients with AD compared to the control, indicating a perturbed metabolic pathway of vitamin B12. A separate role of MMA is in the biosynthesis of pyrimidines (pyrimidine metabolism), the propanoate metabolism and the synthesis of valine, leucine and isoleucine.

2-hydroxyglutaric acid, a widely used marker for gliomas [23], is naturally produced by 2-ketoglutaric or 2-oxoglutaric in the butanoate metabolism. Abnormal accumulation of 2-hydroxyglutarate is observed in hydroxyglutaric acidurias, an inborn metabolic error characterized by neurometabolic manifestations. In the present study, 2-hydroxyglutaric acid was statistically significantly higher in the ADs group compared to the control. Although the effect of elevated 2-hydroxyglutaric acid in nerve cells has not been deciphered, several links have been proposed, including the promotion of oxidative damage, myelin degradation and the disturbance of nerve cells in energy metabolism [24].

2-hydroxyisobutyric acid or a-hydroxyisobutyric was found to be statistically significantly increased in patients with ADs compared to the control. According to the general concept, 2-hydroxyisobutyric acid is not an endogenous metabolite but is a byproduct of methyl tert-butyl ether, which can be obtained from the environment and is rapidly excreted from the body. However, recent studies indicate that 2-hydroxyisobutyric acid is associated with human health [25,26] while suggesting that its levels are strongly correlated with endogenous metabolites indicating an endogenous origin [27].

3-hydroxybutyric acid or b-hydroxybutyric acid is a member of the ketone bodies (including also acetoacetic acid), which are formed in the liver from fatty acids in periods of fasting and carbohydrates restrictive diets. Ketone bodies can also be formed after intensive exercise, excessive alcohol consumption or type 1 diabetes. Their natural role is to fuel the citric acid cycle to provide energy, or they can be converted into long-chain fatty acids in the brain. The group of ADs had elevated levels of 3-hydroxybutyric acid, which reached statistical significance after FDR adjustment (Figure 2). Elevated levels of 3-hydroxybutyric acid are a clinical marker of ketoacidosis and disturbed insulin sensitivity in fasted and diabetic patients. Therefore, markers of insulin sensitivity, including 3-hydroxybutyric acid and 2-hydroxybutyric acid, may have application in ADs due to the close interrelationship between insulin elevated levels causing lipolysis reduction and excessive fatty acids storage that results in local inflammation [28].

4-hydroxyphenylpyruvic acid (4-HPPA) is a keto acid involved in the tyrosine catabolic pathway. In particular, 4HPPA can be biosynthesized from L-tyrosine through its interaction with tyrosine aminotransferase. Subsequently, 4HPPA can be converted into homogentisic acid, mediated by 4-hydroxyphenylpyruvate dioxygenase. Homogentisic acid contributes to the regulation of the tocopherol and tocotrienol biosynthetic pathway (Vitamin E biosynthesis). Moreover, 4-HPPA, via its multistep conversion into 4-hydroxybenzoate, is related to the ubiquinone biosynthetic pathway. Ubiquinone, also known as coenzyme Q, is a coenzyme family, with coenzyme Q10 being the most common form in humans, present primarily in the mitochondria as a component of the electron transport chain and aerobic cellular respiration [29]. Vitamin C, which is involved in the oxidative degradation of tyrosine, is associated with the activity of HPPD, suggesting that 4-HPPA would be a valuable marker for vitamin C bioavailability and uptake [30]. In the present study, 4-HPPA was found to be significantly decreased in patients with AD, indicating an abnormal metabolism of tyrosine and possible association with vitamin C bioavailability. In a previous study, 4-HPPA was found to be associated with diabetes [31] and autoimmune thyroiditis [32].

Enrichment analysis was performed for the 28 metabolites. The butanoate metabolism pathway was found to be the most important metabolic pathway since succinic acid, 2-ketoglutaric acid, 2-hydroxyglutaric acid and 3-hydroxybutyric acid were identified in the pathway, followed by the propanoate metabolism.

Butanoate or butyrate metabolism is responsible for the metabolism of butyric acid, which is formed under bacterial fermentation of carbohydrates to succinic acid for the citric acid cycle, the formation of ketone bodies (3-hydroxybutyric and acetoacetate), or short-chain lipids. Based on our results, butanoate metabolism is substantially altered, which can be seen by the altered levels of metabolites directly involved in the metabolism of butyrate (namely 2-hydroxyglutaric acid and succinic acid) but also the related pathways.

Propanoate metabolism is responsible for the metabolism of propionate through a metabolic reaction pathway where propionate is converted to propionyl-CoA and then to MMA under the activity of MMA-CoA mutase and vitamin B12 and then to succinyl-CoA and succinic acid, which is further used in the citric acid cycle. The origin of propionic acid is the intestinal microflora, while propionyl-CoA can derive from fatty acids or amino acids metabolism. Collectively, in our combination of metabolites, MMA, succinic, methylcitric, and 2-hydroxybutyric participate in the propanoate metabolism. Our findings suggest that patients with AD have significant disturbance in propanoate metabolism.

Aiming to explore the potency of organic acids as predictive biomarkers for ADs, three predictive models were developed using as input the absolute concentrations of organic acids, age, gender, BMI, alcohol consumption and physical exercise levels. PCA, a variable reduction method, was used to identify similarities and differences among the AD group and the control group, reaching 66.8% predictive accuracy. Binary logistic regression model analysis of the Bonferroni corrected metabolites identified two metabolites and two lifestyle variables as being determinant for the model. 2-hydroxyisobutyric and 2-hydroxybutyric were negatively and positively associated with the absence of AD, reaching statistical significance (*p* < 0.0001 and *p* = 0.015), respectively. In line with our previously published work, exercise was positively associated with the absence of AD (*p* < 0.0001), while alcohol consumption was negatively associated with the absence of AD (*p* = 0.002). Besides which, ANN analysis of organic acids and lifestyle factors showed that the most important predictors were the following, in order of importance: pyroglutamic acid, 2-hydroxyglutaric, 2-hydroxyisobutyric, 2-hydroxybutyric and methylmalonic acid. It should be noted that the “relative importance” depicted in Figure 6 of ANN variables refers only to the presence or the lack of predictive information for each variable and does not represent any particular information concerning the statistical significance of the included variables, which is given a priori in any ANN model. Predictive accuracy values from the binary logistic regression model and the ANN were comparably reaching 74.9% and 66.8% overall score, respectively, though ANN was more potent in the discrimination of the AD group (92.6%).

A strength of this study is the integration of targeted metabolomic analysis of selected organic acids that participate in key cellular metabolic pathways with advanced statistics and artificial intelligence. Targeted metabolomics, the quantitative analysis of known metabolites in human biofluid samples, is a sensitive and low-cost method that allows the determination and measurement of a priori selected metabolites. As discussed elsewhere, the advantage of targeted metabolomics over untargeted metabolomics is that it can have application in the validation of potent predictive biomarkers facilitating their application in clinical practice [16]. ADs, as with many other chronic diseases, pre-exist years before symptoms appear, and unfortunately, diagnosis is performed only once the disease is established and has resulted in partly tissue or organ damage. Consequently, there is a big challenge for physicians to manage the symptomatology of ADs and slow down their progression to extend life expectancy and improve their quality of life [33,34]. Proper use of valid biomarkers, in addition to the regular check-up, would potentiate the prediction and subsequent early diagnosis of ADs.

The present study has some limitations. The analysis of ADs as a group may hamper the disease-specific metabolic profile that could have a diagnostic value. However, as discussed elsewhere, ADs share common features, including genetic loci and molecular pathways, suggesting that a grouped analysis would provide valuable information on the common metabolic disturbance [18,35]. Additionally, comorbidities are substantially frequent in ADs, and in some cases, an underlying AD might be undiagnosed or unnoticed for years, hampering the single-disease study analysis. Recent evidence also suggests that different ADs such as myasthenia gravis and rheumatoid arthritis have metabolic overlap enhancing the view of common immunometabolic pathways among ADs [32]. As has been described in the related literature [13,18,36], sample size determination remains a complex step in metabolomic studies since this type of data is correlated and very sensitive. In statistical theory, there are some attempts to identify significant effects via the determination of the adequate sample size in order to capture patient heterogeneity, type I and type II errors. Nonetheless, in practical research, there are restrictions on the availability of training samples, and usually, researchers include only 30–50 patients per group. Despite the fact that in the present work the number of participants was well above this number, some over-fitting issues still remain, and our results should be interpreted with caution. To limit this type of bias, we investigated several ANN models with more complex structures (two hidden layers), but the overfitting was even higher in this case. Hence, we used an ANN model with a simple structure (one hidden layer), and we also split our data into three different data sets (training, test, holdout) to measure the level of overfitting. The difference between the predictive accuracy of “Test” dataset (79.2%) vs. “Holdout” dataset (66.7%) represents the magnitude of overfitting. This model, despite these issues, could serve as a starting point and a benchmark for future work in this field.

Statistical analysis indicates that although the percentage of correct case groups is more than 90%, this model cannot satisfactorily predict the control group and thus, the predictive power of the models is rather limited. This effect has been previously observed by our research team when analyzing total fatty acids in an ADs group, and the results were comparable. As a general comment, we need to highlight that the selection of control groups for predictive and analytic purposes is a common issue in case-control studies. In our case-control study, the aim was to investigate the differential expression and predictive value of organic acids for Ads, having as a hypothesis that these are different in case and controls. However, absolute metabolite values are very sensitive to diet and lifestyle factors (as also shown in our study), thus making the control sample diverse and overlapping with the ADs group. However, as can be observed from our analysis, ADs are associated with OAs levels despite the lifestyle-associated fluctuations of metabolites.

From a statistical standpoint, even though we have conducted advanced non-linear techniques to investigate the differences between the two study arms, the inclusion criteria of healthy individuals should be considered in future metabolomics studies. Hence, selection bias is an important issue in the question at hand since the ideal control group would comprise a random sample from the general population that gave rise to the cases [37]. In our case, we included individuals with no diagnosed disease following the inclusion criteria. However, a portion of the sample may have a different metabolic profile (compared to the rest of the control group), possibly related to diet, lifestyle or underlying metabolic complication, which cannot be pre-assessed with established clinical markers. This is depicted in the large standard deviations of the significantly dysregulated metabolites.

To overcome this barrier in the field, large studies covering the above-mentioned factors affecting the metabolites followed by longitudinal studies have to be conducted to optimize the control group criteria for these types of studies by defining the healthy metabolic group.

## 4. Methods

### 4.1. Study Design

This study was conducted from retrospective data of subjects that have visited the Health clinics of Autoimmune and Chronic Diseases in Athens, Greece, during the period of 26/11/2018 to 28/11/2019 as a part of a monocentric exploratory case-control study. An exploratory case-control study is a type of analysis that aims to identify putative risk factors for disease indicating directions of association and not to provide an inference-based or hypothesis-testing study reflecting accurate magnitudes of statistical associations. Selected autoimmune diseases were multiple sclerosis, autoimmune thyroiditis, psoriasis, vitiligo, lupus, rheumatoid arthritis, inflammatory bowel diseases and other less common autoimmune diseases (for a full list, see Table S1).

The study was undertaken based on 392 participants for both arms, for whom there were detailed electronic records. A total of 241 diagnosed patients with an autoimmune disease were included, while 151 subjects were assigned to the group of healthy controls. Data used in this study included participants’ age, gender, BMI, organic acids measurements, the quantity of consumed alcohol, minutes/week of physical exercise. Eligible participants for the study were men and women of 18–60 years old, with 18.5 > BMI < 30 without being diagnosed with an acute condition or chronic disease (other than AD). Athletes, obese, pregnant or lactating women were excluded from both groups, and for the control group, individuals taking medication and/or supplements were not included.

For the AD group, inclusion criteria for each condition were:

RA: ACR/EULAR 2010 Rheumatoid Arthritis Classification Criteria [38]
IBD: the Lennard-Jones diagnostic criteria for Ulcerative colitis and Crohn’s disease [39]
PSO: The presence of chronic psoriasis plaque and the (Psoriasis Area and Severity Index) PASI score was used to assess the severity of the disease.
THY: Diagnosis and assessment of disease severity were performed by evaluating the levels of the thyroid gland hormones T3 T4 and TSH, and images of the thyroid gland ultrasound.
MS: Revised McDonald 2010 diagnostic criteria [40]

The present study was performed in compliance with the 1964 Helsinki declaration, or comparable ethical standards and participants after they were informed regarding the processing of their anonymized data in accordance to the EU General Data Protection Regulation (GDPR) and they signed informed consent. The study has been approved by the scientific board of the “Health clinic for autoimmune and chronic diseases” and the Ethics Committee of the University of Crete (approval no. A.P. 63_22032019).

All the available data of the present analysis was collected by trained administrative staff via the electronic system of the clinic. Two members of the research team screened the available sample for administrative and typographical errors but none were found. Outliers in each variable were clinically assessed but without any deletion since the sample was considered representative. The percentage of missing values was less than 1% and the imputation was undertaken using the mean values in each variable. Additionally, for the false discovery analysis rate, we normalized the main variables to remove biases via the normalization by median and Pareto scaling (Metaboanalyst) (Figure S4).

### 4.2. Chemicals

N,O,-bis-(trimethylsilyl) trifluoroacetamide (BSTFA) 1% trimethylchlorosilane (TMCS) (both from Supelco Bellefonte, PA, USA), 2-ketocaproic and tropic acids as internal standards (both from Sigma Aldrich (St. Louis, MO, USA)), Hydroxylamine hydrochloride.

### 4.3. Sample Preparation

For the organic acids identification and absolute concentration, our previously published methodology was applied [15]. Briefly, urine samples were collected from fasted patients in a sterilized urine collection container and stored at −80 °C upon analysis. Liquid-liquid extraction was performed to extract the organic acids for urine samples after mixing the sample with 2-ketocaproic and tropic acid as internal standards. Hydroxylamine hydrochloride was added to perform the oxidation of 2-keto acids, and N,O,-bis-(trimethylsilyl) trifluoroacetamide (BSTFA) containing 1% trimethylchlorosilane (TMCS) was added for the organic acids conversion to corresponding trimethylsilyl (TMS) ethers, required to impart volatility. Volatile TMS esters derived from organic compounds are separated in the gas-chromatography, which contains an immobilized, non-polar stationary phase. Detection is performed using an electron impact mass spectrometer in the scan mode with a mass range of 50 and 550 *m/z*. Obtained spectra are compared with published spectra for the compounds of interest to achieve identification. The absolute quantification of organic acids is performed using the calibration curves of standard compounds to internal standard ratios. Concentrations were normalized to creatinine. Τhe quality assurance of the Organic acids’ methodology was assessed by participation in the following Quality control schemes of European Research Network for Diagnosis of Inherited disorders of Metabolism (ERNDIM): Qualitative urine Organic acids and Quantitative urine Organic acids.

### 4.4. Statistical Analysis

In the present work, statistical procedures were conducted using IBM SPSS 22 software [41] and the r-project software [42]. The chi-square test represents a common statistical technique used in Metabolomics, investigating the hypothesis that categorical variables in the population are independent. To investigate any potential statistically significant correlation between the presence of any type of AD and sex, a chi-squared test with continuity correction was conducted. A normality test was conducted to investigate the assumption that biomarkers follow the normal distribution, based on QQ-plots graphics. The term “Q-Q” stands from “Quantile-Quantile”. It represents a graphical tool that can test the hypothesis that the distribution of the available data set does not differ significantly from any theoretical distribution. A univariate descriptive statistical analysis based on median as a central tendency measure was used to analyze differences across organic compounds based on the Mann–Whitney U test [43]. To limit possible false-positive conclusions, a Bonferroni correction was conducted as a standard approach of this type of analysis [44]. Principal Component Analysis (PCA) was also conducted to reproduce the data information with the use of new and fewer variables, which correspond to a linear combination of the originals. Then, a logistic model was designed based on a reduced set of optimum principal components of the original predictors via the step-wise backward selection method. The use of this method has been described in the related literature [45]. Additionally, we employed an artificial neural network (ANN) framework as a predicted model of ADs based on previous scientific [46,47]. The present ANN model represents a feed-forward neural network that was trained with the error backpropagation algorithm and is in line with a recently published model in which the reader can also find a brief introduction to the subject [18]. The accuracy of the model was assessed using the Receiver Operating Characteristic (ROC) for all the models [48].

### 4.5. Matching Analysis

It should be noted that in case-control studies, the matching of patients from both groups is the commonly used method, and its use aims to adjust for confounding at the design stage. The term “matching” refers to a statistical method that aims to finds similar characteristics against which the effect of the treatment can be assessed. Matching has several advantages and also disadvantages, and thus, there are considerations concerning its proper use. In the usual case, matching between case and controls takes place using the Propensity Score Matching (PSM) to estimate causal treatment effects [49]. The term “propensity score” indicates the probability of receiving the treatment given the observed covariates. Nonetheless, frequently, matching produces similar results with un-matched analysis, without any particular gain in efficiency [50]. To explore these possibilities, we conducted an analysis based on one of the most common techniques, “the nearest neighbor algorithm,” concerning age and BMI for both groups. There was a small gain in terms of efficiency and, thus, the research team decided to conduct an unmatched analysis (for details, please see Figure S1).

### 4.6. Enrichment and Pathway Analysis

Quantitative enrichment analysis (QEA) and Pathway Analysis were performed using MetaboAnalyst 4.0 [51]. We aimed to identify metabolite change patterns in ADs compared to the control and correlate them with known human metabolic networks. A concentration table was used with levels of OAs of patients with ADs compared to the control group, they were normalized by a median, log-transformed and Pareto scaled before analysis. Normalized data were then analyzed using the database Kyoto Encyclopedia Genes and Genomes (KEGG).

## 5. Conclusions

ADs are chronic conditions that have a substantial impact on the quality of life of patients, and treatment efficacy highly depends on the stage of the disease and the metabolic state of the patient. As such, identification of people that are at high risk of developing ADs could be an invaluable tool for physicians and patients to make metabolic interventions, including lifestyle and diet, to improve their risk. The current study identified nine differentially expressed metabolites in the AD group compared to the control participating mainly in the TCA and the glutathione cycle providing evidence that patients with ADs have reduced energy production and detoxification mechanisms. Besides which, ANN analysis identified MMA, 2-hydroxyglutaric acid and 2-hydroxybutyric acid to be potent biomarkers for the prediction of ADs, thus further studies would validate these findings.

Overall, these findings indicate that the TCA cycle, the glutathione cycle and the ketone body metabolism pathways are significantly affected in patients with ADs and metabolites involved in these pathways can be potent biomarkers in the unraveling of ADs metabolic pathways and the prediction of ADs presence.

## Figures and Tables

**Table 1:** Comparative organic acids analysis in the autoimmune diseases (ADs) group compared to control. Concentrations of organic acids are expressed as mmol/mol Creatinine. Non-Parametric Mann-Whitney test with Bonferroni Correction, Ho: The distribution of characteristics is the same between the groups. Bold indicates that the variables are considered statistically significant (*p* < 0.05) based on Bonferroni correction. 5-HIAA: 5-Hydroxyindoloacetic acid, 4-HPPA: 4-Hydroxyphenypyruvic acid.

	AD		Control		
	Mean ± SD	Median	Mean ± SD	Median	*p*-Value
Citric acid	88.45 ± 66.17	72.70	96.2 ± 75.7	75.70	>0.90
Isocitric acid	5.04 ± 4.99	4.00	5.21 ± 3.76	4.30	>0.90
2-ketoglutaric acid	11.99 ± 11.54	8.90	15.86 ± 16.57	11.20	0.145
**Succinic acid**	**3.07 ± 7.27**	**1.40**	**4.91 ± 13.82**	**2.00**	**<0.001**
Fumaric acid	0.04 ± 0.27	0.00	0.07 ± 0.31	0.00	>0.90
**Malic acid**	**0.40 ± 0.86**	**0.00**	**0.66 ± 0.63**	**1.00**	**<0.001**
3-hydroxy3-methylglutaric acid	2.17 ± 1.75	1.70	2.19 ± 2.13	1.80	>0.90
Lactic acid	7.88 ± 9.63	5.60	16.81 ± 75.43	7.00	0.232
Pyruvic acid	7.76 ± 6.04	6.60	8.61 ± 6.4	6.80	>0.90
3-hydroxybutyric acid	9.14 ± 54.47	0.00	5.44 ± 16.3	1.00	0.638
**Pyroglutamic acid**	**19.04 ± 16.90**	**16.70**	**23.97 ± 16.29**	**21.10**	**<0.001**
3-hydroxyisovaleric acid	10.25 ± 10.52	7.80	13.98 ± 15.29	9.90	0.087
**Methylmalonic acid**	**0.63 ± 0.97**	**0.00**	**0.95 ± 0.87**	**1.00**	**<0.001**
Homovanillic acid	2.12 ± 1.63	1.70	2.57 ± 2.4	2.10	0.29
5-HIAA	2.69 ± 3.01	2.10	3.51 ± 5.52	2.50	0.725
4 Hydroxyphenylacetic acid	11.40 ± 13.41	7.50	10.96 ± 8.86	8.00	>0.90
Orotic acid	0.01 ± 0.16	0.00	0.01 ± 0.11	0.00	>0.90
**2-Hydroxyglutaric acid**	**2.53 ± 1.71**	**2.20**	**1.95 ± 4.23**	**1.30**	**<0.001**
Glycolic acid	22.68 ± 17.91	18.70	26.86 ± 23.11	22.30	>0.90
Oxalic acid	4.66 ± 3.55	4.00	5.95 ± 4.54	5.00	>0.90
Glyceric acid	2.04 ± 7.56	0.00	1.52 ± 4.08	1.30	>0.90
**2-hydroxy isobutyric acid**	**4.75 ± 2.81**	**4.20**	**2.79 ± 3.94**	**0.00**	**<0.001**
**2-hydroxy butyric acid**	**0.16 ± 0.77**	**0.00**	**0.39 ± 0.96**	**0.00**	**<0.001**
Ethylmalonic acid	1.64 ± 2.26	1.20	1.9 ± 1.9	1.40	0.812
Methylsuccinic acid	0.34 ± 0.86	0.00	0.17 ± 0.54	0.00	>0.90
Suberic acid	0.08 ± 0.55	0.00	0.1 ± 0.39	0.00	>0.90
**Methylcitric acid**	**0.11 ± 0.31**	**0.00**	**0.27 ± 0.45**	**0.00**	**<0.001**
**4HPPA**	**0.55 ± 0.88**	**0.00**	**0.79 ± 0.76**	**1.00**	**<0.001**

**Table 2:** Association of the presence of autoimmune disease with patient’s characteristics and organic acids levels; Dependent Variable: Absence of autoimmune disorder; Binary Logistic Regression Model; *p* < 0.05 are shown in bold. 4-HPPA: 4-Hydroxyphenypyruvic acid LCI: Lower Confidence Interval; UCI: Upper Confidence Interval.

	B	St Error	Exp (B)	95% LCI	95% UCI	*p*-Value
Succinic acid	0.018	0.012	1.018	0.994	1.044	0.147
Malic acid	0.059	0.181	1.060	0.744	1.512	0.747
Pyroglutamic acid	0.015	0.010	1.015	0.995	1.036	0.151
Methylmalonic acid	0.005	0.180	1.005	0.706	1.431	0.976
2-Hydroxy-glutaric acid	−0.069	0.048	0.933	0.850	1.024	0.145
**2-hydroxy isobutyric acid**	**−0.180**	**0.044**	**0.835**	**0.766**	**0.910**	**0.000**
**2-hydroxy butyric acid**	**0.420**	**0.172**	**1.521**	**1.086**	**2.131**	**0.015**
Methylcitric acid	0.389	0.332	1.476	0.769	2.831	0.241
4HPPA	0.188	0.175	1.207	0.857	1.700	0.281
Female	−0.450	0.271	0.638	0.375	1.085	0.097
Age	−0.009	0.011	0.991	0.969	1.014	0.440
**No Exercise**	**−0.985**	**0.262**	**0.373**	**0.223**	**0.624**	**0.000**
**No Alcohol**	**0.830**	**0.264**	**2.294**	**1.367**	**3.849**	**0.002**
BMI	−0.009	0.027	0.991	0.939	1.045	0.735
Constant	0.352	0.837	1.422			0.674

**Table 3:** Association of the presence of autoimmune disease with the Principal Components Dependent Variable: Absence of autoimmune disorder; Binary Logistic Regression. LCI: Lower Confidence Interval; UCI: Upper Confidence Interval.

	B	St Error	Exp (B)	95% LCI	95% UCI	*p*-Value
Factor 1	0.076	0.129	1.079	0.838	1.388	0.557
Factor 2	0.137	0.124	1.146	0.899	1.461	0.270
Factor 3	0.454	0.203	1.575	1.059	2.344	0.025
Factor 4	−0.611	0.126	0.543	0.424	0.695	0.000
Factor 5	0.297	0.129	1.346	1.045	1.735	0.022
Factor 6	0.061	0.112	1.063	0.853	1.324	0.586
Factor 7	−0.031	0.125	0.969	0.759	1.239	0.804
Factor 8	−0.185	0.166	0.831	0.600	1.151	0.265
Factor 9	0.047	0.116	1.049	0.836	1.316	0.682
Factor 10	−0.300	0.168	0.741	0.533	1.030	0.074
Male	−0.095	0.251	0.909	0.556	1.487	0.705
Age	−0.014	0.012	0.986	0.964	1.009	0.231
BMI	−0.002	0.027	0.998	0.947	1.052	0.952
Exercise	0.993	0.261	2.698	1.617	4.502	0.000
Alcohol	−0.850	0.261	0.427	0.256	0.713	0.001
Constant	−0.202	0.779	0.817			0.795

**Table 4:** Classification Table for Artificial Neural Network-Organic Acids.

	Predicted			
		Case	Control	% Correct
**Training**	Case	164	7	95.9%
	Control	49	51	51.0%
	Overall Percent	78.6%	21.4%	79.3%
**Testing**	Case	42	3	93.3%
	Control	12	15	55.6%
	Overall Percent	75.0%	25.0%	79.2%
**Holdout**	Case	25	2	92.6%
	Control	15	9	37.5%
	Overall Percent	78.4%	21.6%	66.7%

## Data Availability

The dataset presented in this study is available from the corresponding author upon reasonable request.

## Supplementary Materials

The following are available online at https://www.mdpi.com/2218-1989/10/12/502/s1, Figure S1. Diagnostics for a matched case-control analysis. Figure S2. p-value distribution and False Discovery Rate computation by Q-value for both arms Histogram of adjusted p-values (Q-values) based on False Discovery Rate (FDR) using all the p values generated by the bivariate two-tailed Spearman Rank Correlation. Figure S3. Box plot of main variables by group (untransformed scale). Figure S4. Normalization by Median and Pareto scaling for main variables. Table S1. List of diseases grouped as “other” in the group of patients with autoimmune diseases, Table S2. Comparative organic acids analysis in the ADs group compared to control after imputation of values <LOD. Table S3. Baseline characteristics of the case and control group, Table S4. Component Score Coefficient Matrix, Table S5. Model Parameters for the Artificial Neural Network of Organic Acids.
