# Supplementary material for: Prediction of Autoimmune Diseases by Targeted Metabolomic Assay of Urinary Organic Acids
Source: Metabolites. 2020 Dec 8;10(12):502. doi: 10.3390/metabo10120502 (PMC7764183; doi:10.3390/metabo10120502)
Supplement: Supplementary file 1 [file metabolites-10-00502-s001.zip › Supplementary/Rev Supplementary.docx]

| **List of diseases** |
| --- |
| Asthma |
| Raynaud syndrome |
| Ankylosing spondylitis |
| Polyarthritis |
| Psoriatic arthritis |
| Scleroderma |
| Spoldyloarthritis |
| Osteoarthritis |
| Osteoporosis |
| Dermatitis - Eczema |
| Lupus |
| Autoimmune Polymyositis |
| Primary biliary cholangitis |
| Sjogren's syndrome |
| Alopecia areata |

**Table S1.** List of diseases grouped as “other” in the group of patients with autoimmune diseases.

|  | **AD** | | **Control** | |  |
| --- | --- | --- | --- | --- | --- |
|  | **Mean+SD** | **Median** | **Mean+SD** | **Median** | **p-value** |
| Citric acid | 87.71+65.77 | 72.30 | 96.3+75.43 | 75.80 | >0.90 |
| Isocitric acid | 5.05+4.99 | 4.00 | 5.22+3.73 | 4.30 | >0.90 |
| 2-ketoglutaric acid | 12.07+11.55 | 9.10 | 15.86+16.56 | 11.20 | 0.197 |
| **Succinic acid** | **3.14+6.62** | **1.40** | **5.04+13.77** | **2.00** | **<0.001** |
| Fumaric acid | 0.72+0.17 | 0.71 | 0.73+0.15 | 0.71 | 0.643 |
| **Malic acid** | **0.91+0.65** | **0.71** | **0.95+0.35** | **1.00** | **<0.001** |
| 3-hydroxy3-methylglutaric acid | 2.25+1.66 | 1.70 | 2.28+2.03 | 1.80 | >0.90 |
| Lactic acid | 7.9+9.65 | 5.60 | 16.82+75.42 | 7.00 | 0.225 |
| Pyruvic acid | 7.8+6.03 | 6.60 | 8.61+6.39 | 6.80 | >0.90 |
| 3-hydroxybutyric acid | 9.54+54.51 | 0.71 | 5.7+16.2 | 1.00 | 0.496 |
| **Pyroglutamic acid** | **19.02+16.91** | **16.60** | **24+16.23** | **21.10** | **0.004** |
| 3-hydroxyisovaleric acid | 10.34+10.5 | 7.80 | 14.01+15.25 | 9.90 | 0.106 |
| **Methylmalonic acid** | **1.01+0.76** | **0.71** | **1.17+0.65** | **1.00** | **0.002** |
| Homovanillic acid | 2.11+1.53 | 1.70 | 2.6+2.36 | 2.10 | 0.198 |
| 5-HIAA | 2.73+2.97 | 2.10 | 3.53+5.5 | 2.50 | 0.711 |
| 4 Hydroxyphenylacetic acid | 11.37+13.39 | 7.50 | 10.97+8.84 | 8.00 | >0.90 |
| Orotic acid | 0.71+0.1 | 0.71 | 0.71+0.03 | 0.71 | >0.90 |
| 2-Hydroxyglutaric acid | 2.56+1.65 | 2.20 | 2.27+4.08 | 1.30 | **<0.001** |
| Glycolic acid | 22.78+17.91 | 18.70 | 26.88+23.08 | 22.30 | >0.90 |
| **Oxalic acid** | **4.67+3.36** | **4.00** | **5.98+4.48** | **5.00** | **0.040** |
| Glyceric acid | 2.41+7.5 | 0.71 | 1.79+3.98 | 1.30 | 0.140 |
| **2-hydroxy isobutyric acid** | **4.81+2.73** | **4.30** | **3.14+3.69** | **0.71** | **<0.001** |
| **2-hydroxy butyric acid** | **0.81+0.65** | **0.71** | **0.91+0.77** | **0.71** | **<0.001** |
| Ethylmalonic acid | 1.81+2.15 | 1.20 | 2+1.81 | 1.40 | 0.748 |
| Methylsuccinic acid | 0.89+0.67 | 0.71 | 0.8+0.34 | 0.71 | 0.156 |
| Suberic acid | 0.76+0.45 | 0.71 | 0.75+0.22 | 0.71 | >0.90 |
| **Methylcitric acid** | **0.73+0.09** | **0.71** | **0.78+0.14** | **0.71** | **0.001** |
| **4HPPA** | **0.98+0.64** | **0.71** | **1.04+0.53** | **1.00** | **0.004** |

**Table S2.** Comparative organic acids analysis in the ADs group compared to control after imputation of values< LOD. Concentrations of organic acids are expressed as mmol/mol Creatinine. Non-Parametric Mann-Whitney test with Bonferroni Correction, Ho: The distribution of characteristics is the same between the groups. Bold indicates that the variables are considered statistically significant (p < 0.05) based on Bonferroni correction. 5-HIAA: 5-Hydroxyindoloacetic acid, 4-HPPA: 4-Hydroxyphenypyruvic acid; concentration values below the Level of Detection was replaced with LOD/√2=0.707 whereby LOD=1mmol/mol Crea

|  | **Case**  **(n=243)** | **Control (n=151)** |
| --- | --- | --- |
| ^*^Age (Mean + SD) | 44,5+11,3 | 42,8+10,0 |
| ^*^Female (%) | 69,5 | 62,9 |
| ^*^BMI (Mean + SD) | 25,3+4,7 | 25,0+4,1 |
| ^a**^ Exercise n (%) | 136 (56,0) | 115 (75,2) |
| ^b**^ Alcohol n (%) | 106 (43,6) | 40 (26,5) |
| THY n (%) | 44 (18,1) | 0 |
| RA n (%) | 4 (1,6) | 0 |
| IBD n (%) | 20 (8,2) | 0 |
| MS n (%) | 24 (9,5) | 0 |
| PSO n (%) | 16 (6,6) | 0 |
| AD n (%) | 1 (0,4) | 0 |
| VIT n (%) | 5 (2,1) | 0 |
| OTHER n (%) | 38 (15,6) | 0 |

**Table S3.** Baseline characteristics of the case and control group. BMI: Body Mass Index, THY: Thyroid Autoimmune Disease, RA: Rheumatoid Arthritis, IBD: Inflammatory Bowel Disease, MS: Multiple Sclerosis, PSO: Psoriasis, VIT: Vitiligo; “^a^” indicates Exercise >3times per week; “^b^” indicates alcohol consumption of 3 glasses of wine per week; “^*^” indicates p-value > 0,05; “^**^” indicates p-value<0,001.

|  | 1 | 2 | 3 | 4 | 5 | 6 | 7 | 8 | 9 | 10 |
| --- | --- | --- | --- | --- | --- | --- | --- | --- | --- | --- |
| Citric | .105 | .127 | -.094 | .108 | .084 | .005 | -.074 | -.108 | -.005 | .019 |
| 2-ketoglutaric | .058 | .220 | -.008 | .072 | -.014 | -.039 | -.087 | -.056 | -.006 | .014 |
| Malic | -.012 | .275 | .008 | -.215 | -.033 | -.020 | -.016 | -.014 | -.069 | -.042 |
| Isocitric | .197 | -.014 | -.057 | .065 | -.051 | .194 | -.008 | -.028 | -.033 | .059 |
| Succinic | .027 | -.022 | .027 | -.030 | .087 | .716 | -.035 | .000 | -.003 | .023 |
| Fumaric | -.061 | .369 | -.001 | .052 | .031 | .020 | -.017 | .050 | -.009 | -.097 |
| 3-hydroxy 3-methylglutaric | .012 | -.136 | -.100 | .013 | .090 | -.219 | -.128 | -.181 | -.552 | .065 |
| lactic | -.005 | -.067 | .440 | -.004 | -.054 | .058 | -.003 | -.163 | .070 | -.002 |
| Pyruvic | .164 | -.048 | .220 | .044 | -.030 | .024 | -.071 | -.057 | .086 | -.003 |
| 3-hydroxybutyric | -.048 | .064 | .066 | -.018 | -.097 | .175 | .064 | .143 | -.598 | -.062 |
| Pyroglutamic | .181 | -.037 | .039 | -.006 | .025 | -.099 | -.023 | -.048 | -.025 | .020 |
| 3 hydroxyisovaleric | .019 | -.065 | .217 | .002 | .110 | -.200 | -.056 | .138 | -.110 | .062 |
| Methylmalonic | .184 | -.071 | .064 | -.183 | -.011 | -.063 | -.019 | .077 | -.013 | .032 |
| Homovanillic | .032 | .027 | -.148 | .089 | .308 | .001 | .094 | -.097 | -.100 | .022 |
| 5-HIAA | .182 | .019 | -.109 | -.091 | -.069 | -.137 | .271 | -.053 | -.156 | -.097 |
| 4 Hydroxyphenylacetic | .103 | -.180 | -.086 | -.070 | .169 | .157 | -.045 | -.075 | -.109 | .201 |
| orotic | -.066 | -.065 | .009 | -.017 | -.039 | .010 | -.028 | .019 | .001 | .678 |
| 2-Hydroxyglutaric | .056 | .207 | -.062 | .179 | -.084 | .121 | .096 | -.061 | .163 | .233 |
| glycolic | -.070 | .022 | .214 | .039 | .279 | -.097 | -.167 | .206 | .171 | .016 |
| oxalic | -.094 | .009 | .015 | -.033 | .513 | .083 | .028 | .014 | .037 | -.083 |
| Glyceric | .058 | -.015 | -.107 | -.024 | .011 | .002 | -.019 | .771 | -.015 | .020 |
| 2-hydroxy isobutyric | -.061 | .092 | .058 | .385 | .038 | -.076 | .167 | .188 | -.066 | .215 |
| 2-hydroxy butyric | -.068 | .136 | .334 | .013 | -.043 | .056 | .134 | .047 | -.165 | -.032 |
| ethylmalonic | .249 | .032 | -.039 | .061 | -.067 | .044 | .000 | .152 | .121 | -.146 |
| methylsuccinic | .036 | .054 | -.075 | .329 | .158 | -.130 | .011 | -.059 | .097 | -.291 |
| suberic | -.051 | .029 | -.025 | .003 | -.069 | .036 | -.813 | .033 | -.004 | .002 |
| methylcitric | -.022 | .089 | -.030 | -.382 | .136 | -.079 | .166 | .116 | .030 | .009 |
| 4OHPPyr | .007 | .166 | -.038 | -.167 | .070 | -.141 | .010 | -.093 | .110 | .189 |
| % Variance | 22.1 | 7.3 | 6.3 | 6.1 | 4.7 | 4.4 | 4.2 | 3.7 | 3.5 | 3.3 |
| % Cumulative | 22.1 | 29.5 | 35.8 | 41.9 | 46.6 | 51.0 | 55.2 | 58.9 | 62.4 | 65.8 |

**Table S4.** Component Score Coefficient Matrix. Rotation Method: Oblimin with Kaizer Normalization

|  | Hidden Layer 1 | | | | | | | | Output Layer | |
| --- | --- | --- | --- | --- | --- | --- | --- | --- | --- | --- |
|  |  |  |  |  |  |  |  |  |  |  |
|  | H(1:1) | H(1:2) | H(1:3) | H(1:4) | H(1:5) | H(1:6) | H(1:7) | H(1:8) | [Group=0] | [Group=1] |
| (Bias) | -1.161 | 1.287 | -1.710 | -.338 | -.208 | .296 | 1.579 | -1.198 |  |  |
| [Exercise=0] | -.649 | 1.164 | -.273 | .825 | -.827 | 1.323 | 2.129 | -.380 |  |  |
| [Exercise=1] | -.197 | -0.233 | -1.695 | -1.418 | .340 | -0.806 | -0.615 | -.696 |  |  |
| [Alcohol=0] | .212 | 0.846 | -.303 | -1.150 | .768 | -0.607 | 0.560 | .528 |  |  |
| [Alcohol=1] | -.727 | -0.464 | -1.197 | .636 | -.857 | 1.414 | 0.687 | -1.419 |  |  |
| [Sex=F] | -.600 | 2.178 | -.615 | -.556 | -.403 | 0.251 | 0.490 | -.118 |  |  |
| [Sex=M] | -.018 | -0.892 | -.491 | -.110 | .786 | 0.157 | -0.009 | -1.309 |  |  |
| Succinic | -.255 | -0.920 | -.497 | -.136 | -.580 | -0.256 | -0.383 | -.669 |  |  |
| Malic | -.231 | 0.124 | -.830 | -1.155 | .499 | -0.832 | -0.295 | .195 |  |  |
| Pyroglutamic | .329 | -1.712 | -.405 | -1.132 | -.726 | -1.225 | -0.208 | -.516 |  |  |
| Methylmalonic | -.311 | -1.978 | 1.389 | .410 | 1.487 | -0.344 | 2.090 | -1.213 |  |  |
| @2Hydroxyglutaric | 1.958 | 2.224 | -2.620 | -.268 | .665 | 0.010 | 3.084 | -1.537 |  |  |
| @2hydroxyisobutyric | .867 | 0.423 | -3.098 | 1.068 | -1.814 | 0.867 | -0.279 | .318 |  |  |
| @2hydroxybutyric | -.599 | -0.585 | 1.144 | 1.001 | -.189 | -0.704 | -0.131 | 1.021 |  |  |
| methylcitric | -.994 | 0.641 | -.186 | 1.113 | .881 | -0.701 | -1.394 | 1.433 |  |  |
| @4OHPPyr | -.820 | -1.732 | .143 | 1.101 | 3.777 | -1.511 | 0.420 | -1.282 |  |  |
| (Bias) |  |  |  |  |  |  |  |  | -0.158 | 0.150 |
| H(1:1) |  |  |  |  |  |  |  |  | -2.273 | 2.325 |
| H(1:2) |  |  |  |  |  |  |  |  | 2.116 | -2.149 |
| H(1:3) |  |  |  |  |  |  |  |  | -3.090 | 3.073 |
| H(1:4) |  |  |  |  |  |  |  |  | 1.589 | -1.519 |
| H(1:5) |  |  |  |  |  |  |  |  | -2.238 | 2.289 |
| H(1:6) |  |  |  |  |  |  |  |  | -1.173 | 1.277 |
| H(1:7) |  |  |  |  |  |  |  |  | 2.481 | -2.446 |
| H(1:8) |  |  |  |  |  |  |  |  | -1.916 | 1.896 |

**Table S5.** Model Parameters for the Artificial Neural Network of Organic Acids


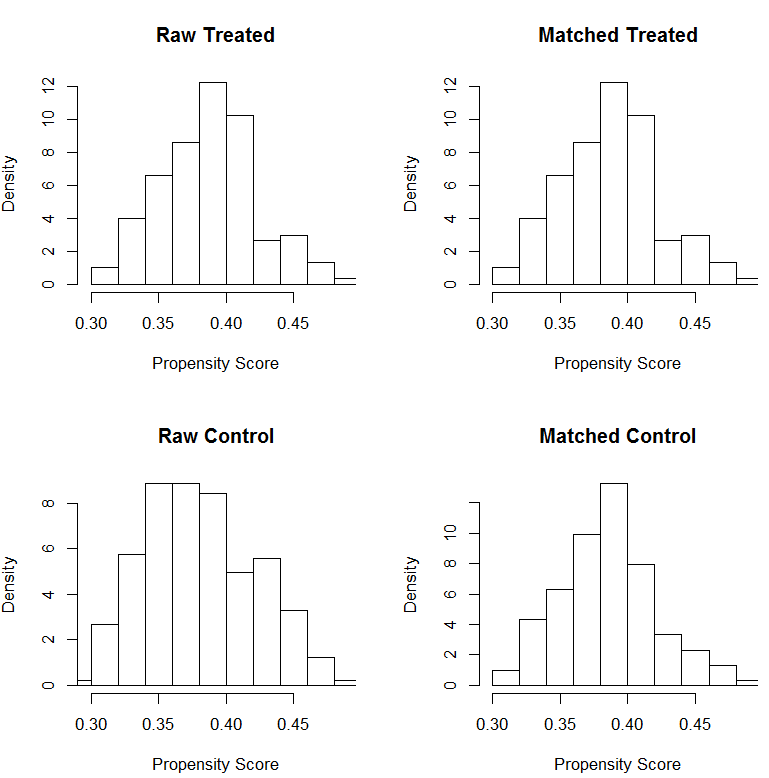

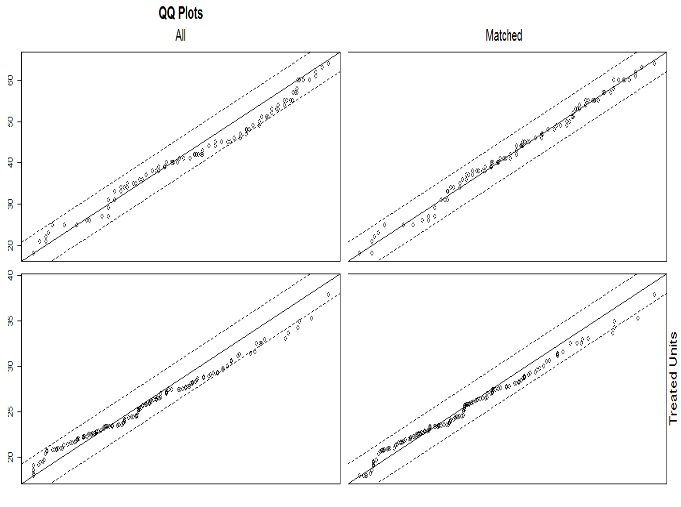
**Figure S1** Diagnostics for a matched case-control analysis

# *Cases Controls*

# Figure S2. P-value distribution and False Discovery Rate computation by Q-value for both arms Histogram of adjusted p-values (Q-values) based on False Discovery Rate (FDR) using all the p values generated by the bivariate two-tailed Spearman Rank Correlation; the dashed horizontal line denotes the pre-specified significance level of 0.05;

### FigureS3. Box plot of main variables by group (untransformed scale)


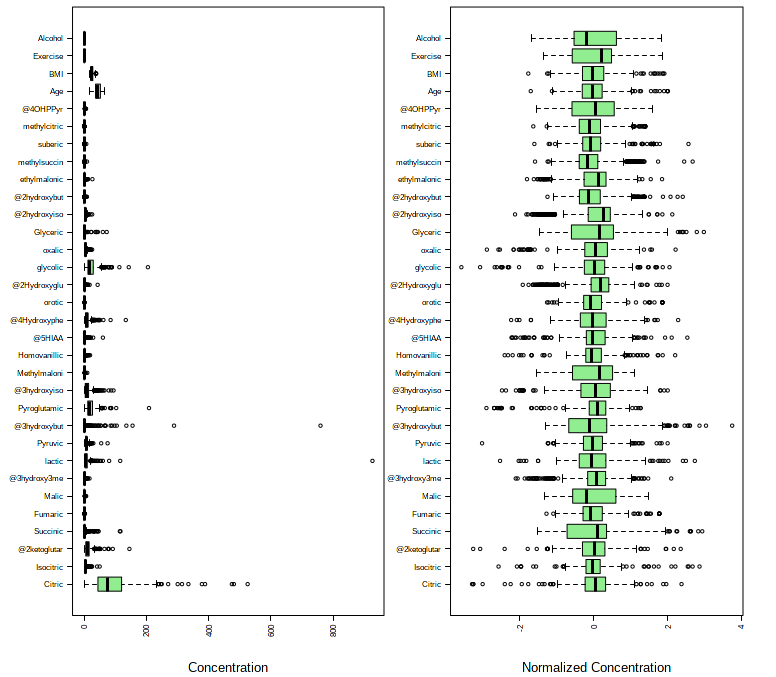


**Figure S4** Normalization by Median and Pareto scaling for main variables
